# A syndrome of severe intellectual disability, hypotonia, failure to thrive, dysmorphism, and thinning of corpus callosum maps to chromosome 7q21.13‐q21.3

**DOI:** 10.1111/cge.14143

**Published:** 2022-05-05

**Authors:** Daniel Halperin, Nadav Agam, Maher Hallak, Miora Feinstein, Max Drabkin, Yuval Yogev, Ohad Wormser, Eitan Shavit, Libe Gradstein, Ilan Shelef, Aanalia Mijalovsky, Hagit Flusser, Ohad S. Birk

**Affiliations:** ^1^ The Morris Kahn Laboratory of Human Genetics National Institute for Biotechnology in the Negev, Ben‐Gurion University of the Negev Beer‐Sheva Israel; ^2^ Department of Ophthalmology Soroka University Medical Center and Clalit Health Services, Ben‐Gurion University of the Negev Beer‐Sheva Israel; ^3^ Department of Imaging, Soroka University Medical Center Faculty of Health Sciences, Ben‐Gurion University of the Negev Beer‐Sheva Israel; ^4^ Zusman Child Development Center, Division of Pediatrics Soroka University Medical Center, Ben‐Gurion University of the Negev Beer‐Sheva Israel; ^5^ Genetics Institute Soroka University Medical Center Beer‐Sheva Israel

**Keywords:** craniofacial dysmorphism, disease‐associated locus, intellectual disability, novel syndrome

## Abstract

Six individuals of consanguineous Bedouin kindred presented at infancy with an autosomal recessive syndrome of severe global developmental delay, positive pyramidal signs, unique dysmorphism, skeletal abnormalities, and severe failure to thrive with normal birth weights. Patients had a profound intellectual disability and cognitive impairment with almost no acquired developmental milestones by 12 months. Early‐onset axial hypotonia evolved with progressive muscle weakness, reduced muscle tone, and hyporeflexia. Craniofacial dysmorphism consisted of a triangular face with a prominent forehead and midface hypoplasia. Magnetic resonance imaging (MRI) demonstrated thinning of the corpus callosum and paucity of white matter. Genome‐wide linkage analysis identified a single ~4 Mbp disease‐associated locus on chromosome 7q21.13‐q21.3 (LOD score>5). Whole‐exome and genome sequencing identified no nonsynonymous pathogenic biallelic variants in any of the genes within this locus. Following the exclusion of partially resembling syndromes, we now describe a novel autosomal recessive syndrome mapped to a ~4Mbp locus on chromosome 7.

## Graphical abstract

We describe a novel autosomal recessive syndrome mapped to a ~4Mbp locus on chromosome 7; Six individuals of consanguineous kindred presented at infancy with a severe global developmental delay, positive pyramidal signs, unique dysmorphism and failure to thrive, with MRI demonstrating thinning of the corpus callosum and white matter paucity.

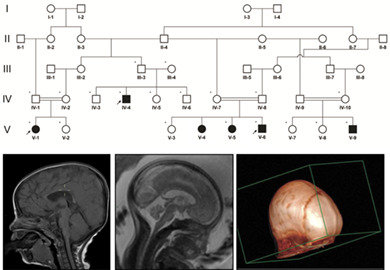

## 1 INTRODUCTION

The Bedouin community in southern Israel is unique in its high consanguinity and inbreeding, with a high‐incidence of monogenic diseases.^1^ Some pathogenic variants remain elusive, likely representing mutations in untranslated and noncoding functional elements.^2^ We now describe an autosomal recessive syndrome of global developmental delay with profound intellectual disability, severe failure to thrive (FTT), axial hypotonia, and positive pyramidal signs with unique dysmorphism and skeletal abnormalities, mapping to chromosome 7q21.13‐q21.3, with no definite identifiable pathogenic coding variants within this locus. The unique clinical presentation and exclusion of partially resembling syndromes suggest a novel disease.

## 2 METHODS

### 2.1 Clinical phenotyping

Six individuals were studied following informed consent and approval of Soroka University Medical Center Internal Review Board. Phenotyping was determined by pediatric neurologists, ophthalmologists and geneticists.

### 2.2 Linkage and sequence analysis

Genome‐wide linkage analysis of 19 family members of a single clan, and whole‐exome sequencing (WES) for three affected individuals (IV:4, V:4, V:1; Figure 1) were performed. Whole‐genome sequencing (WGS) was performed for IV:4. WES and WGS data were analyzed using QIAGEN's Ingenuity Variant Analysis software (http://www.qiagenbioinformatics.com/ingenuity-variant-analysis; QIAGEN Redwood City, California, USA), excluding variants with allele frequency ≥1% in the Genome Aggregation Database (gnomAD, https://gnomad.broadinstitute.org/), or variants appearing in a homozygous state in our in‐house WES database of 400 controls. Of the remaining variants, we selected only those segregating within the family as expected for autosomal recessive heredity. Multipoint LOD score was calculated using SUPERLINK_ONLINE_SNP1.1 (http://cbl-hap.cs.technion.ac.il/superlink-snp/).

**FIGURE 1:**
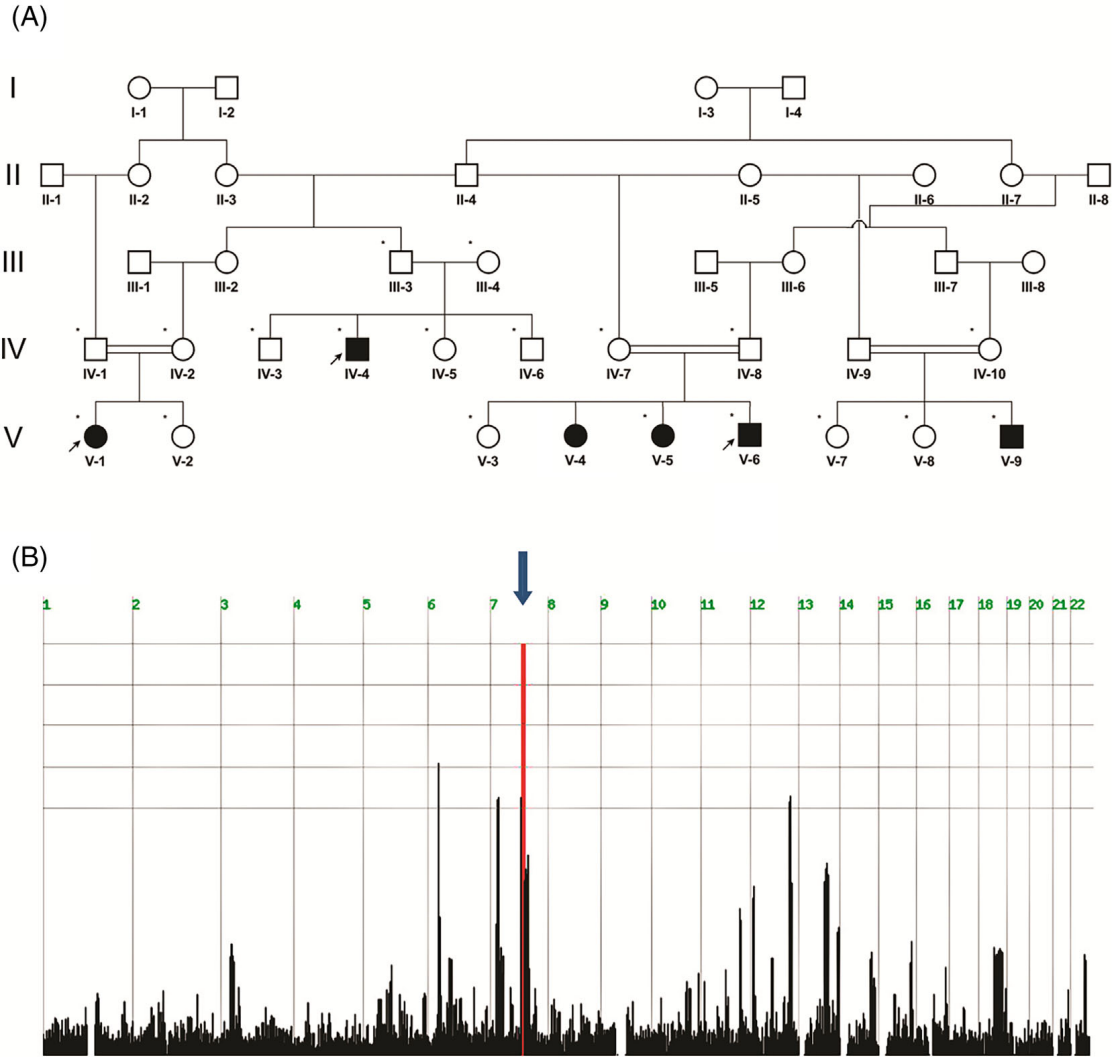
Pedigree and linkage analysis. (A) The studied kindred. As can be seen, there are at least two families involved in the analysis, that is, descendants of I:1, I:2, and I:3, I:4. These families are related as individual IV:1 is heterozygous for the 7q21.13‐q21.3 locus. Genome‐wide linkage analysis included 19 family members, denoted by asterisks. WES was performed for three patients, denoted by arrows. (B) Homozygosity scores. Genome‐wide SNP distributions were collected for 19 family members. The distribution of homozygous regions was determined using HomozygosityMapper (http://www.homozygositymapper.org/). Blue arrow indicates the single homozygous locus on chromosome 7q21.13‐q21.3 between rs6952664 and rs13234589, shared by affected individuals. [Colour figure can be viewed at wileyonlinelibrary.com]

## 3 RESULTS

### 3.1 Clinical characterization

Six patients of consanguineous Bedouin kindred (Figure 1A) presented (birth to 6 months) with an autosomal recessive syndrome of global developmental delay, profound mental retardation, axial hypotonia, positive pyramidal signs, unique dysmorphism, and skeletal abnormalities (Table 1). Regarding the pedigree, there are at least two families of the same clan involved in the analyses, that is, descendants of I:1, I:2, and I:3, I:4. To note, patient III:4, although allegedly not related to the family, is also a direct descendant of the lineage.

**TABLE 1:** Detailed clinical phenotypes of affected individuals

	V:9	V:4	IV:4	V:5	V:1	V:6
Gender	Male	Male	Male	Female	Female	Female
Gestational age	term	term	36	term	term	term
Birth weight (g)	3060	2680	2400	3200	2730	3100
OFC (cm)	38	Normal	Normal	Normal	34.5	35
Age of presentation (months)	8	3.5	3	Birth	4	Birth
Global developmental delay	+	+	+	+	+	+
Intellectual disability	+	+	+	+	+	+
Poor feeding	+	+	+	+	+	+
FTT	+	+	+	+	+	+
Positive pyramidal signs	+	+	+	+	n/a	+
Convulsions	−	+	−	−	−	−
Axial hypotonia	+	+	+	+	+	+
Intention tremor	+	−	−	−	−	−
Choreiform hand movements	+	+	+	+	n/a	+
Craniofacial dysmorphism						
Triangular face	+	+	+	+	+	+
Low set ears	+	+	+	+	+	+
Pinched nose	+	+	+	+	+	+
Trigono/Plagio‐cephaly	Metopic	+	+	+	n/a	+
Sialorrhea	+	+	−	−	+	−
Brain MRI						
Thinning, corp. Callosum	+	+	+	+	+	+
Ventriculomegaly	+	+	−	+	+	n/a
BEH	−	+	+	−	−	n/a
Hearing impairment	+	+	+	+	+	n/a
Ocular involvement						
Visual difficulties	Ocular fixation present at 14 months	Absent ocular fixation at 19 months	Follows lights, not objects at age 33 months	n/a	Delayed fixation development (appeared at 18 months)	Ocular fixation present at 12 months
Strabismus	+	+	−	−	+	−
Nystagmus	−	+intermittent	−	+	+intermittent	+
Hypermetropia/Astigmatism	+/−	−/+	n/a	n/a	+/+	+/+
VEP, ERG	n/a	Both normal	ERG: normal VEP: reduced amplitude	n/a	n/a	n/a
Developmental aphasia	+	+	+	+	+	+
Dysphagia	+	+	+	+	+	+
Limb involvement						
Muscle tone	Low	Low	Low	Low	normal	Low
Muscle wasting	−	−	−	−	−	−
Contractures	−	−	−	−	−	−
Hyporeflexia	+	+	+	+	n/a	+
Cardiac abnormalities	−	Systolic murmur	PDA, Pulmonic stenosis	PDA, mitral and tricuspid regurgitation SVT	−	−
Skeletal deformities						
Clinodactyly	+	+	−	+	n/a	+
Over‐riding toes 2–3	+	+	+	+	n/a	+
Syndactyly	n/a	+	+	+	n/a	+
GE reflux	−	+	+	+	+	n/a
Congenital hypothyroidism	−	−	+	+	−	−
Cryptorchidism	+	+	−	−	−	−
Additional findings	Pyloric stenosis, post. Fossa arachnoid cyst	OSA		Cerebellar dysgenesis, Germinal matrix cysts, Congenital CMV infection		

Abbreviations: BEH, benign external hydrocephalus; ERG, electroretinogram; FTT, failure to thrive; GE, gastroesophageal; mo, months; n/a, data not available; OFC, occipitofrontal circumference; OSA, obstructive sleep apnea; PDA, patent ductus arteriosus; SVT, supraventricular tachycardia; VEP, visual evoked potential; y, years.

**FIGURE 2:**
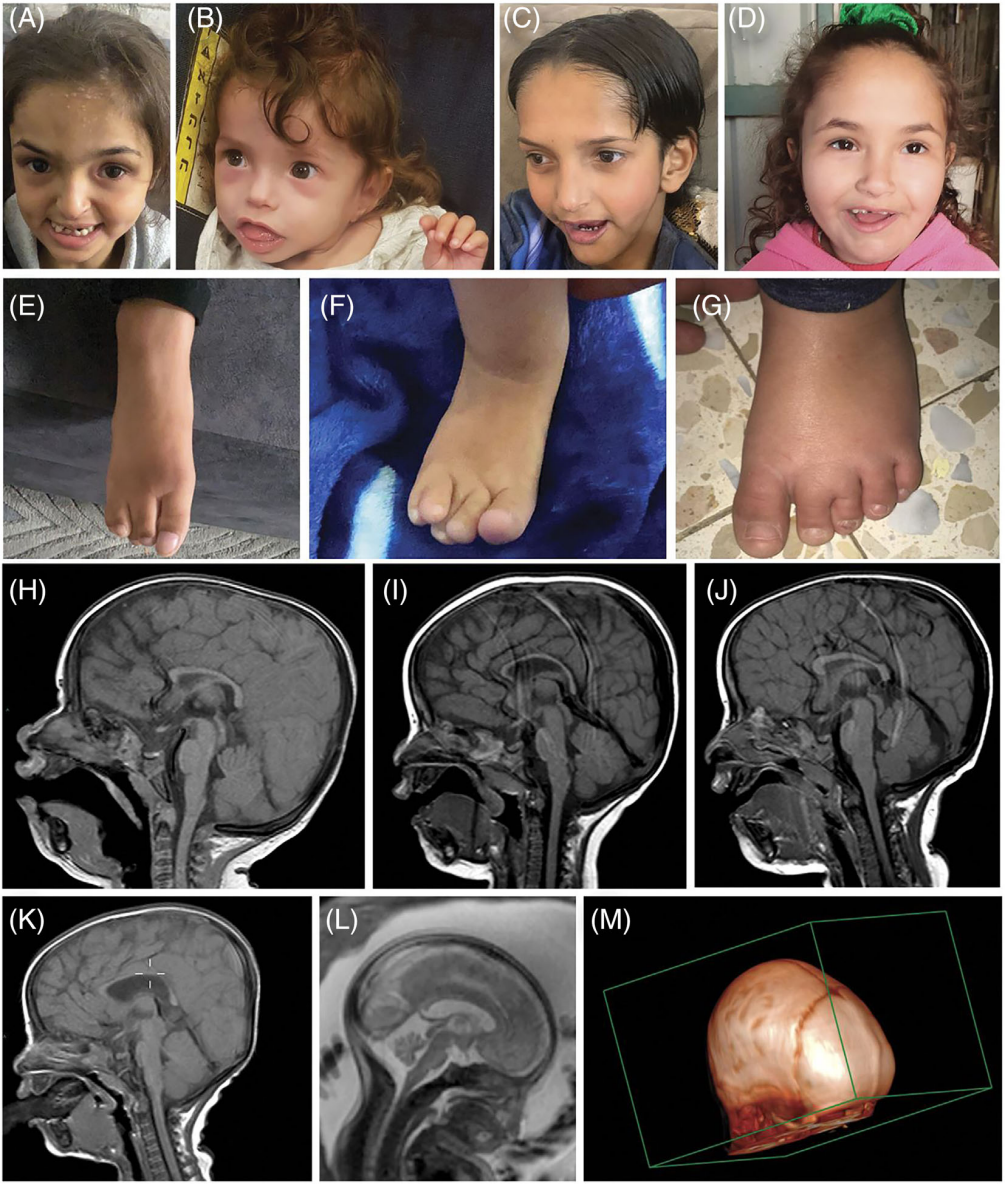
Clinical characterization. (A–D) Patients V:5, V:6, IV:4, and V:1, respectively. Craniofacial dysmorphism was apparent with normal to borderline small head circumference, characteristic triangular face with prominent forehead, slightly posterior‐rotated low‐set ears, and small chin. (E–G) Patients V:5, V:6, and V:1, respectively: over‐riding toes, with clinodactyly or syndactyly. (H–K) Brain MRI of patients IV:4, V:4, V:5, and V:9, respectively. (L) Brain MRI of patient V:6 in‐utero. All affected individuals showed thinning of corpus callosum with paucity of white matter. (M) Metopic stenosis of patient V:9. [Colour figure can be viewed at wileyonlinelibrary.com]

All patients were born at term following uneventful pregnancies (birth weights 2400‐3500 g) with subsequent postnatal feeding difficulties and dysphagia, culminating in severe short stature and FTT. Craniofacial dysmorphism was apparent in all, with normal (in most) to borderline small head circumference, triangular face with prominent forehead, slightly posterior‐rotated low‐set ears, small narrow‐bridged nose, and small chin, somewhat reminiscent of facial gestalt elements of Russel Silver Syndrome (RSS), Zellweger and to a lesser extent ‐ Seckel syndrome (Figure 2A–D). Three patients were diagnosed with trigonocephaly (V:4, V:5, and V:6), one with plagiocephaly (IV:4), and one with metopic synostosis (V:9). Affected individuals had a profound intellectual disability and cognitive impairment with almost no acquired developmental milestones by 12 months: no visual or verbal communication and only limited ambulation. Early‐onset axial hypotonia evolved with progressive muscle weakness, reduced muscle tone, and hyporeflexia, affecting proximal and distal extremities. Choreiform hand movements and positive pyramidal signs were evident. Patients had over‐riding toes 2–3, with clinodactyly or syndactyly (Figure 2E–G). At least five patients suffered from congenital hearing problems, evident through brainstem evoked response audiometry.

Ophthalmic examination, performed on five patients who could cooperate with the exam, showed poor visual function, not following light or objects before 1 year; ocular fixation developed between 12 and 33 months. One patient could not follow visual stimuli by 19 months and had roving eye movements. Four patients presented with nystagmus (intermittent in two). Strabismus (with horizontal and vertical components) was evident in two patients. Refractive errors were detected in four patients, three of whom had hypermetropia (2.50–7.50 diopters), and three had astigmatism. One patient displayed anterior chamber abnormalities, including posterior embryotoxon of the cornea (prominent Schwalbe's line), uneven iris pigmentation, and iris heterochromia. Posterior eye segment exam in four patients showed normal retina and optic nerve. Flash visual evoked potentials (VEP) and full‐field electroretinogram, obtained under anesthesia in two patients, demonstrated normal function of the retinal photoreceptors and typical latency of VEP responses, suggesting normal conduction of the visual stimuli to the brain; however, response amplitude was reduced in one patient, likely reflecting paucity of brain white matter.

Brain MRI of all affected individuals in early childhood (Figure 2H–L) showed thinning of the corpus callosum and paucity of white matter,^3^ with ventriculomegaly in four. MRI of patient V:9, in addition to metopic stenosis (Figure 2M), demonstrated posterior fossa arachnoid cyst with mass effect, treated with ventriculo‐peritoneostomy.

Other pathologies, manifested by some of the patients, included gastroesophageal reflux (4/6), cryptorchidism (2/3), patent ductus arteriosus (2/6), congenital hypothyroidism (2/6), systolic murmur (1/6), convulsions (1/6), tongue protrusion with sialorrhea (2/6), pulmonary stenosis (1/6), micrognathia (1/6) and pyloric stenosis (1/6). It is unclear whether these manifestations are part of the syndrome. Blood pH, lactate, creatine phosphokinase, amino acids, and urinary organic acids were within normal limits for all patients. Plasma very‐long‐chain fatty acids (VLCFA), carnitine, and acylcarnitine, as well as blood tests of liver enzymes, were also unremarkable. Blood pyruvate and ketone body levels were mildly elevated.

### 3.2 Genetic analysis

Karyotype and chromosomal microarray (CMA) analyses were normal. Standard methylation / uniparental disomy/microdeletion testing for RSS (chromosome 11 and 7 loci) was negative. Genome‐wide linkage analysis, testing 19 family members (including affected individuals IV:4, V:5, V:6, V:9, V:1), identified only one locus of homozygosity shared by the affected individuals, with heterozygosity demonstrated by all unaffected parents: a ~ 4Mbp segment on chromosome 7q21.13‐q21.3 between rs6952664 and rs13234589 (Figure 1B), segregating as expected within the studied kindred (LOD score 5.01 at rs17865021). Following filtering of WES data of patients IV:4, V:1, and V:6 for normal variants as aforementioned, no homozygous or compound heterozygous nonsynonymous variants within the 7q21.13‐q21.3 locus were found to segregate within the pedigree as expected for recessive heredity. Notably, no variants were found in any of the genes previously associated with RSS (*CDKN1C*, *IGF2*, *PLAG1*, and *HMGA2*; OMIM #180860*)*, Seckel syndrome (*ATR, RBBP8*, *CENPJ*, *CEP152*, *CEP63*, *NIN*, *DNA2*, *TRAIP*, *NSMCE2*; OMIM #21600) or Zellweger syndrome (*PEX 1*, *PEX6*, *PEX12*, *PEX26*, *PEX10*, *PEX2*, *PEX5*, *PEX13*, *PEX16*, *PEX3*, *PEX19*, *PEX14*, *PEX11β*; ΟΜΙΜ#214 100). A synonymous *PEX1* variant (NM_000466.3; c.2271C>T; p.F757F), within the 7q21.13‐q21.3 locus, segregated within the kindred as expected with possible disease association. However, this variant is common in the Bedouin population (32 heterozygous carriers of 400 samples) with homozygosity in six controls. Another variant segregating within this locus, in *AKAP9* (NM_005751.4; c.5228 T > G; p.V1801G), demonstrated heterozygosity in 7% of the Bedouin cohort and homozygosity in eight controls. Finally, a combined NGS analysis of the affected patients (including WGS data of IV:4) identified no homozygous or compound heterozygous likely pathogenic variants in other regions outside the 7q21.13‐q21.3 locus that segregate as expected for recessive heredity.

## 4 DISCUSSION

We delineate a novel autosomal recessive syndrome of intellectual disability, severe global developmental delay and axial hypotonia, with dramatic FTT yet normal birth weight, distinct facial dysmorphism, congenital hearing problems and delayed development of ocular fixation, aberration in toe structure, and thinning of the corpus callosum with a paucity of white matter.

The disease phenotype is somewhat reminiscent of RSS–both in the facial gestalt and FTT;^4^ however, the normal birth weight, lack of asymmetry, and extreme global developmental delay differ significantly from RSS. Moreover, extensive molecular studies ruled out findings previously associated with RSS.^4^

Another possible differential diagnosis is Zellweger syndrome–with similar components of facial dysmorphism, hypotonia, severe global developmental delay, and possible findings in corpus callosum imaging.^5^ However, no other stigmata of Zellweger (liver dysfunction, adrenal insufficiency, renal oxalate stones on ultrasound, or bone stippling on X‐rays) were seen. Moreover, plasma VLCFA was normal, and no variants were found in genes previously associated with Zellweger syndrome.^6^ Notably, a synonymous variant in *PEX1*,^7^ found in affected individuals, is a normal variant in the Bedouin cohort.

Some elements of the dysmorphism and the thin corpus callosum resemble Seckel syndrome.^8^ However, the significant microcephaly of Seckel syndrome mostly lacks in the kindred we describe, and the severe global developmental delay contrasts with the majority of cases of Seckel syndrome.^9^ Moreover, no sequence variants were found in genes previously associated with Seckel syndrome. Notably, congenital hearing problems and delayed visual development with aberrant findings in ophthalmologic examination, that are not found in any of the syndromes in the differential diagnosis, were evident in the affected individuals we describe.

The pathogenic variant remains elusive, likely affecting one of the 28 genes within the 7q21.13‐q21.3 locus, 10 of which were previously associated with clinical phenotypes (Table S1). Most notable are *PEX1* (discussed above), *CDK6* ‐ associated with primary microcephaly,^10^ *SAMD9* ‐ associated with autosomal dominant MIRAGE syndrome,^11^ and *VPS50* ‐ associated with a neurodevelopmental disorder with microcephaly and seizures, but with no other features of the discussed syndrome.^12^ Filtering our WGS data for noncoding variants revealed a highly conserved promoter variant in *CALCR* (NM_001164737.2; c.‐795C>T, CADD>20). However, this variant was found in two healthy controls. The 7q21.13‐q21.3 locus also harbors four microRNA genes, with no currently known clinical significance related to the syndrome we present. No additional noncoding/splicing likely pathogenic variants were found through our genome analysis to segregate in the kindred as expected for a disease‐causing mutation.

Altogether, our findings demonstrate a consistent phenotype of severe intellectual disability, global developmental delay, hypotonia, and FTT, with unique facial dysmorphism as well as thinning of the corpus callosum, delineating a novel autosomal recessive syndrome mapped to a ~ 4Mbp locus on chromosome 7.

## CONFLICTS OF INTEREST

The authors declare no conflicts of interest.

## AUTHOR CONTRIBUTIONS

Genetic and molecular studies: Daniel Halperin, Nadav Agam, Maher Hallak, Miora Feinstein, Max Drabkin, Yuval Yogev. Ohad Wormser, Eitan Shavit, Ohad S. Birk. Clinical characterization: Daniel Halperin, Ilan Shelef, Libe Gradstein, Aanalia Mijalovsky, Hagit Flusser, Ohad S. Birk. Writing the manuscript: Daniel Halperin, Ohad S. Birk. Ohad S. Birk initiated and supervised the project. Approved by authors.

### PEER REVIEW

The peer review history for this article is available at https://publons.com/publon/10.1111/cge.14143.

## ETHICS STATEMENT

Soroka Medical Center IRB ‐ 5071G.

## Supporting information


**Table S1** Candidate genes found within the segregating locus on chromosome

## Data Availability

The data that support the findings of this study are available from the corresponding author upon reasonable request.

## References

[1] Markus B , Alshafee I , Birk OS . Deciphering the fine‐structure of tribal admixture in the Bedouin population using genomic data. Heredity (Edinb). 2014;112:182‐189. 24084643 10.1038/hdy.2013.90 PMC3907104

[2] Scacheri CA , Scacheri PC . Mutations in the non‐coding genome. Curr Opin Pediatr. 2015;27:659‐664. 26382709 10.1097/MOP.0000000000000283 PMC5084913

[3] Andronikou S , Pillay T , Gabuza L , et al. Corpus callosum thickness in children: an MR pattern‐recognition approach on the midsagittal image. Pediatr Radiol. 2015;45:258‐272. 25173405 10.1007/s00247-014-2998-9

[4] Wakeling EL , Brioude F , Lokulo‐Sodipe O , et al. Diagnosis and management of silver–Russell syndrome: first international consensus statement. Nat Rev Endocrinol. 2017;13:105‐124. 27585961 10.1038/nrendo.2016.138

[5] Steinberg SJ , Raymond GV , Braverman NE , Moser AB . Zellweger spectrum disorder. GeneReviews®[Internet]. University of Washington; 2017.

[6] Klouwer FCC , Berendse K , Ferdinandusse S , Wanders RJA , Engelen M . Zellweger spectrum disorders: clinical overview and management approach. Orphanet J Rare Dis. 2015;10:151. 26627182 10.1186/s13023-015-0368-9 PMC4666198

[7] Crane DI , Maxwell MA , Paton BC . PEX1 mutations in the Zellweger spectrum of the peroxisome biogenesis disorders. Hum Mutat. 2005;26:167‐175. 16086329 10.1002/humu.20211

[8] Faivre L , le Merrer M , Lyonnet S , et al. Clinical and genetic heterogeneity of Seckel syndrome. Am J Med Genet. 2002;112:379‐383. 12376940 10.1002/ajmg.10677

[9] Verloes A , Drunat S , Gressens P , Passemard S . Primary autosomal recessive microcephalies and Seckel syndrome spectrum disorders. GeneReviews®[Internet]. University of Washington; 2013. 20301772

[10] Hussain MS , Baig SM , Neumann S , et al. CDK6 associates with the centrosome during mitosis and is mutated in a large Pakistani family with primary microcephaly. Hum Mol Genet. 2013;22:5199‐5214. 23918663 10.1093/hmg/ddt374

[11] Narumi S , Amano N , Ishii T , et al. SAMD9 mutations cause a novel multisystem disorder, MIRAGE syndrome, and are associated with loss of chromosome 7. Nat Genet. 2016;48:792‐797. 27182967 10.1038/ng.3569

[12] Schneeberger PE , Nampoothiri S , Holling T , et al. Biallelic variants in VPS50 cause a neurodevelopmental disorder with neonatal cholestasis. Brain. 2021;144:3036‐3049. 34037727 10.1093/brain/awab206

