# Supplementary material for: A syndrome of severe intellectual disability, hypotonia, failure to thrive, dysmorphism, and thinning of corpus callosum maps to chromosome 7q21.13‐q21.3
Source: Clin Genet. 2022 May 5;102(2):123–9. doi: 10.1111/cge.14143 (PMC9545274; doi:10.1111/cge.14143)
Supplement: Supplementary file 1 — Table S1 Candidate genes found within the segregating locus on chromosome [file CGE-102-123-s001.docx]

**Table S1**

Candidate genes found within the segregating locus on chromosome 7q21.13-q21.3

| **Gene** | **OMIM** | **Phenotype** | **Phenotype OMIM** |
| --- | --- | --- | --- |
| *STEAP1* | #604415 |  |  |
| *STEAP2* | #605094 |  |  |
| *CFAP69* | #617949 | Spermatogenic failure 24 | #617959 |
| LOC101927446 |  |  |  |
| *GTPBP10* | #610920 |  |  |
| LOC101409256 |  |  |  |
| *CLDN12* | #611232 |  |  |
| LOC102723899 |  |  |  |
| *CDK14* | #610679 |  |  |
| *FZD1* | #603408 |  |  |
| *MTERF1* | #602318 |  |  |
| *AKAP9* | #604001 | Long QT syndrome 11? | #611820 |
| *CYP51A1* | #601637 |  |  |
| *LRRD1* |  |  |  |
| *KRIT1* | #604214 | Cavernous malformation of CNS and retina | #116860 |
| miR-1285-1 |  |  |  |
| *ANKIB1* |  |  |  |
| *GATAD1* | #614518 | Dilated cardiomyopathy, 2B? | #614672 |
| *ERVW1* | #604659 |  |  |
| *PEX1* | #602136 | Heimler syndrome; Zellweger; NALD/IRD | #234580; #214100; #601539 |
| *RBM48* |  |  |  |
| *FAM133B* |  |  |  |
| *CDK6* | #603368 | Microcephaly 12, primary? | #616080 |
| *SAMD9* | #610456 | Mirage syndrome; Tumoral calcinosis | #617053; #610455 |
| *SAMD9L* | #611170 | Ataxia-pancytopenia syndrome | #159550 |
| *HEPACAM2* | #614133 |  |  |
| *VPS50* | #616465 | Neurodevelopmental disorder with microcephaly, seizures, and neonatal cholestasis | #619685 |
| *CALCR* | #114131 | Osteoporosis, postmenopausal | #166710 |
| miR-653 |  |  |  |
| miR-489 | #614523 |  |  |
| miR-4652 |  |  |  |
| *TFPI2* | #600033 |  |  |
| LOC105375401 |  |  |  |
| *GNGT1* | #189970 |  |  |
| *GNG11* | #604390 |  |  |
| *BET1* | #605456 |  |  |
